# Education and Training in Global Occupational Health and Safety: A Perspective on New Pathways to Sustainable Development

**DOI:** 10.29024/aogh.2309

**Published:** 2018-10-10

**Authors:** Matteo Paganelli, Egidio Madeo, Ismail Nabeel, Luigi Isaia Lecca, Ilaria Pilia, Sergio Pili, Jacopo Fostinelli

**Affiliations:** 1 University of Brescia, Brescia, IT

## Abstract

The institution of specific Occupational Health and Safety (OHS) training programs open to international trainees from developing countries in some European, American and Asian universities is now a well-established reality. Courses and seminars that focus particularly on this subject, widely varying in approach and duration, have been held for years at these universities; these academic institutions have combined their potential to attract students from developing countries with the scheduling of interesting lectures and training activities, depending on the availability of funds sufficient to cover travel and lodging costs. Interdisciplinarity is the key to the entire program and is its main strength, as the trainees have the opportunity to condense the technical notions and methodological aspects of different disciplines (occupational health, industrial hygiene, safety management, ergonomics) in one course. We firmly believe that these programs are a precious instrument for the training of occupational health professionals from low-income countries, as they are able to address their choices correctly, hopefully achieving the goal of reducing the human costs of development.

## Global health, occupational health, training, sustainable development

The institution of specific Occupational Health and Safety (OHS) training programs open to international trainees from developing countries in some European, American and Asian universities is now a well-established reality. Courses and seminars that focus particularly on this subject, widely varying in approach and duration, have been held for years at these universities; these academic institutions have combined their potential to attract students from developing countries with the scheduling of interesting lectures and training activities, depending on the availability of funds sufficient to cover travel and lodging costs. This allows many trainees to overcome the income gap existing between developing and developed countries, which would otherwise be an insurmountable obstacle. The objective of these courses is to increase the number of occupational health professionals and the quality of their training in low-income countries, in order to contribute to an improvement of the working conditions in such countries, at least where the knowledge and awareness of the best practices and solutions can be useful even if not associated with great expenses and investments, being sufficient in any case to decrease the workers’ exposure to certain workplace hazards [1,2]. One possible criticism of this type of approach relates to the imbalance, in particular from the cultural point of view, which may arise between practices and procedures typical of industrialized countries and the social and economic conditions of developing countries where these practices should be applied. This thesis is clearly supported and confirmed by listing the many expensive technical solutions that have been applied to protect workers in many industries in developed countries and which will probably not be available in developing countries for a while, despite them being considered “state of the art”. These solutions include: negative pressure rooms to protect healthcare personnel from patients affected by sputum positive tuberculosis, retracting needles for injections and punctures, workplaces automated, isolated and equipped with air suction industrial machinery, forklifts and mechanical devices for load handling, isolated and air-conditioned tractors for farming etc. The direct consequence of such an approach would be to assume that the training of occupational health professionals working in developing countries is totally useless, given that most of the required worker safety measures are too expensive and therefore inapplicable. Moreover, it could be considered a failure, from an educational point of view, to export proven technical solutions without simultaneously ensuring a cultural change by addressing the subject of health and safety risks in the workplace. We firmly believe the complete opposite of the provocative statement written above: the definition of *developing country* itself (sometimes euphemistic, we admit) highlights the tendency of a country to tend towards development, whatever the starting point; low-income countries’ economies often grow at extremely high yearly rates as compared to the very low percentages of the often slack economies of the developed world. The main difference in historical perspective between high-income countries with the strongest economies and nations for which the opportunities of development and improvement have been available only for a few years is that the latter could take advantage of the wealth of knowledge (accumulated over hundreds of years) acquired by the former in the field of occupational health and therefore properly manage many of the safety and health issues that industrialization brings [3]. A wealth of knowledge on industrial hygiene and occupational medicine is available and should be applied today in developing countries to support smart and farsighted choices to prevent the continued worldwide repetition of the same development paradigm with its well-known side effects on workers’ health. Asbestos is a fitting example: only the knowledge of the social costs (both human and economic) related to the widespread use of asbestos in the building and manufacturing industry, acquired over the decades, could correctly influence the choices of countries where, due to the low life expectancy at birth, cancer risk is not perceived [4]. The challenges for OHPs working in developing countries are arduous and require the ability to adapt the principles of industrial hygiene and modern occupational medicine to working environments that the Western world sometimes does not even remember: this is the case with health hazards and diseases that followed the geographical shift of low-cost labor-related sectors: respiratory diseases from vegetable textile fibers (byssinosis, bagassosis, etc.) are an example [5]. Additionally, in most contexts, OHPs (often governmental) are very few: under these conditions, they must receive complete and constantly updated training in all the fields of the subject in order to meet the enormous challenges before them [6].

International training programs open to these pioneers are the more useful the more methodological content they provide, as well as information and expertise. Fortunately, today, the information is widely available and free in many countries, thanks to widespread internet connection from mobile devices; what is needed, therefore, is the ability to select the right information and sources. One inevitable benefit of “global” training is the possibility of creating a network of colleagues able to collaborate remotely and provide each other with support and advice. The interweaving of such ties is the root to sustainable development from the point of view of the “human costs” of work and can, in our view, achieve the goal of creating “development shortcuts” by reducing real and perceived distances between countries that are at different points on the same path. It is essential that the didactic approach is based on the active participation of trainees and should feature both theoretical and practical learning through observation and action in working environments; above all, it must also be interdisciplinary [7,8,9]. According to this model, the summer school held every year at the University of Brescia offers a program consisting of classes held by international experts from many different fields of occupational and environmental health sciences: physicians, industrial hygienists, communication specialists and environmental scientists. This is followed by the preparation by each participant, within small workshops, of concise presentations aimed at highlighting aspects of interest for the learner and to provide the teacher with feedback. The formation of working groups, in particular, enables and lays the foundations for direct relationships in future collaborations. In addition to traditional classroom educational activity, the program also offers visits to real industrial plants and workplaces in the region. The sites of interest are selected based on the availability of the companies, in an attempt to offer an overview of the main activities of each industry (agriculture, extraction, manufacturing and services). During the visits, participants are taught by both teachers and OHS staff working at each plant and are invited to evaluate and estimate personally the risk/dangers that characterize each workplace and the solutions adopted and are encouraged to propose improvement measures. Interdisciplinarity is the key to the entire program, and it is its main strength: the analysis of OHS themes evaluated simultaneously from all the points of view that make up its whole spectrum of professions allows an analytical approach that leaves few open questions. Within the context of global learning, it is essential that professional training figures from developing countries have the opportunity to condense as many technical notions and methodological aspects of different disciplines (occupational health, industrial hygiene, safety management and ergonomics) as possible into the program, as they will probably have to manage in their practice complex problems that, in developed countries, are solved by a dedicated specialist (chemical risk, ergonomics, biological risk, safety issues, etc.). Finally, the goal of educational initiatives directed at OHPs working in developing countries is to help them become real “global consultants”, in the geographic sense as well as in terms of the global nature of the knowledge they require: a kind of globalization of prevention [10].
